# Cerebral Rheumatism

**Published:** 1880-07

**Authors:** P. O’Connell


### Article IV.

### Cerebral Rheumatism. By P. O'Connell, m.d.

Five and a half years ago (January, 1875), the *American Journal of the Medical Sciences* published an elaborate paper with the above title, from the pen of Professor Da Costa, of Philadelphia. Cerebral rheumatism must always possess deep interest for practitioners, especially if it may be now and then encountered in a quasi-epidemic form. In the following pages it is intended to make some comments upon this disease and upon the paper named above.

After a careful perusal of the paper, I dissent from the conclusions drawn by the distinguished writer from the study of the twelve cases given by him. To me it seems clear that these were not at all instances of "cerebral rheumatism," as I shall endeavor to show.

The dates in some parts of the notes of the cases related are indefinite and somewhat jumbled. It is not always easy, therefore, to say definitely when certain symptoms first appeared, nor when changes in the treatment were made. The twelve cases embodied in Prof. Da Costa's paper, with the exception of Case IX, which was seen in consultation, and was an instance of chronic muscular rheumatism, were primarily cases of acute articular rheumatism, and, save Case IX, were all treated in the Pennsylvania Hospital. All received the same general, almost identical, treatment.

Dr. Da Costa limits "the term" (cerebral rheumatism) "to cases in which the nervous symptoms are prominent, and appear to constitute the real features of the affection."

"The disorder is not a frequent one, and has been chiefly commented upon in isolated cases. Whether from a distrust of results thus obtained, or from a real rarity of the affection, or whatever the cause, the medical journals and hospital reports may for years be searched in vain for any detailed records of it." *

"It has already been pointed out that uncomplicated rheumatism, however severe it may be, is rarely attended with cerebral disturbance." (Bristowe, First Ed., Pract. Med., page 820.)

That the disease is really rare, is well known. I find statistics of 5,783 cases of acute rheumatism reported by competent, able, fearless men, and no mention is made of any form of cerebral trouble. The reporters are men engaged in active, busy practice, both public and private, with an experience extending over twenty, thirty, and even forty years. Dr. Da Costa believes he has seen no less than twelve cases of this formidable malady in the short space of four years, and attributes this frequency with which the disease is met certain years over others to "some peculiarity of the rheumatic poison, which makes it fix more readily on the brain, as we sometimes see by way of exception the typhus fever poison attacking the intestine, or influenza producing a catarrh of the mucous membrane of the bowel, rather than of that of the respiratory tract." It is just possible, though probably not observed, that Dr. Da Costa has confounded several different and distinct diseases. Such is what, I believe, has happened, and what a perusal of the cases, as detailed in the *American Journal*, will show. I will give here the salient points

* The quotations are from Prof. Da Costa's paper, unless otherwise specified.

of each case; to give them *in extenso*, or even a full abstract of each case, would occupy too much valuable space.

CASE I. Was sick two weeks before admission to hospital on Dec. 26, 1871. Convalesced from the rheumatism. Then albuminuria with suppression of urine followed, and the patient died in a stupor January 9, 1872. The autopsy, in which the brain was not examined, proved the existence of the large, hard kidney—Bright's disease. Clearly death was due to uræmia.

CASE II. Was sick two weeks before admission on February 25, 1871. Urine contained one-sixth albumen, with granular and oily casts. Died March 13, 1871. The autopsy revealed the existence of the large, pale kidney. Lungs congested posteriorly, with a pint of clear fluid in each pleural cavity. No intra-cranial lesion.

CASE III. Was sick ten days before admission on April 23, 1872. No urinary reactions are given. Patient was of feeble mind before admission. Dyspepsia, cough and dullness of right lung posteriorly were noted, as well as some petechiæ on abdomen. Urinated involuntarily in bed. Died April 29, 1872. No organic disease of the brain was found on *post mortem* examination. Meninges seemed congested, "brain firm, pale and anæmic." No other organ was examined.

CASE IV. Was sick four days before admission on January 18, 1872. Was a highly hysterical female. Had cough and "mild, good-natured mental aberration." Defecated and urinated involuntarily in bed. Died March 2, 1872. No disease of brain found on post mortem examination. Other organs healthy.

CASE V. Was four days ill before admission on January 3, 1874. Heart grew very feeble; had oppression in chest, rapid breathing, and "quiet and good-natured," but nearly constant delirium. There were "fine râles and faint friction at lower part of right lung; both lungs were congested." Recovered.

CASE VI. Was ill a week before admission on May 9, 1873. A puny, impoverished subject. Urine albuminous. Died in a stupor June 17, 1873. The brain was not permitted to be examined. The other organs were fairly healthy except the pericardium, which was adherent throughout.

CASE VII. Was ill five weeks before admission on December

23, 1873. There were sonorous râles in posterior chest. Had active delirium one night. Died January 14, 1874. In the autopsy the brain was found anæmic; some hypostatic congestion of the lungs. Other organs healthy.

CASE VIII. Was sick twelve days before admission on March 4, 1872. Was hysterical and had headache, flushed face, prolonged and sobbing respiration at times. Urine not examined during life; some drawn from the bladder by catheter after death was highly albuminous. Micturition occurred involuntarily in bed. Died March 21, 1872 in an unconscious condition, the temperature just before death being 108.2°. No autopsy was permitted.

CASE IX. Chronic muscular rheumatism; seen by Dr. Da Costa in consultation. The notes were lost and the case is quoted from memory. There is insanity in the family; patient became insane, but ultimately recovered her health. Embolism was supposed to be the cause of the particular head trouble (insanity) noticed.

CASE X. Was sick eight days before admission on March 9, 1872. Face was noted pale and puffy, with large red spots on it. A typhoid condition developed. Delirium. Died comatose May 4, 1872. Post mortem: the brain alone was examined and only grossly, when nothing abnormal was seen.

CASE XI. Had "no cerebral symptoms," but the extraordinarily high temperature of 110°. Recovered.

CASE XII. Was ill a week before admission on January 7, 1870, and had treatment before coming into hospital. There was harsh breathing and dullness in posterior inferior thorax. Urine became diminished, albuminous and contained casts. Patient seemed dead once, when all treatment for the rheumatism was suspended and stimulants used very freely. Recovered by dint of stimulants.

"For the morbid anatomy of the affection I must refer to the autopsies in the individual cases. I will only point out that not in one was there any marked congestion of the brain; in tenth the brain is either mentioned as being normal and firm, as in Cases II and IV, or as pale and anæmic, as in Cases III and VII, or without lesion save a slight injection of the vessels of the membranes, as in case X. The brain lesion, or rather the state of the vessels, in Cases III and IV, is most interesting; and so is the acute degenerative disease of the kidneys in Cases I and II. I believe that both these morbid states will be found more frequently if they are closely looked for, and invite the attention of observers to these points. Some of the kidneys in the post mortem inspections here detailed were not examined with the care that, having noticed the changes, I should now bestow on them."

"In none of the autopsies did I meet with meningitis." Yet he admits that meningitis does occur; but he thinks "it is a very rare lesion; and what is called rheumatic meningitis is generally not meningitis at all but cerebral rheumatism with an absence of meningeal lesions.

"The temperature is apt to be high, the joint affection persistent, or even showing signs of increase; the breathing is rapid; the pulse frequent, compressible, and at times irregular. A cardiac difficulty may show itself distinctly as a complication, or again be wholly wanting. In some cases convulsions, in others local palsies happen, or we may have hemiplegia even suddenly developed. But these features are rare; and it is in the wakefulness and restlessness, in the stupor and delerium that we mostly find the signs of how decidedly the brain has become disordered.

"In some of the cases, hurried respiration attracted my attention without anything in the condition of the lungs or heart to account for it; occasionally, however, there is decided congestion of the lower lobes of the lungs."

A petechial eruption was observed in a few cases. "It consists of irregular dull red spots sluggishly influenced by pressure, and which may be perceptible after death. It probably depends on stagnation of the blood in the capillaries from paralysis of certain vaso-motor nerves."

"The delerium is not violent, and is rarely associated with headache. * * * It is generally worse at night; and sometimes has a strange hysterical semblance. The delirium is preceded by wakeful, dreamy nights, is generally mild, and it is during the restless nights that it shows itself most plainly. Though it may be a continuous, it is scarcely ever a fierce delirium, and is not, as a very general rule, linked either to headache, injected eye, or vomiting. It may run a rapid course, delirium or stupor quickly ending in coma, coma in death. But ordinarily it goes on for days, the patient gradually mending or becoming weaker and weaker, and passing, perhaps, into a condition very similar to that of typhod fever, excepting that the bowels are constipated."

Such is the clinical history of this formidable disease as observed by Dr. Da Costa. What is more natural than that, in highly hysterical patients, the delirium should assume "a strange hysterical semblance." This was noted in two females. The temperature is not given in case III. For the eleven others the average maximum temperature is 104°.

The urinary reactions published are scanty and imperfect. In cases I, II and VI, the urine was found decidely albuminous; in case VIII that fluid was not tested during life. After death it "was found to contain a large proportion of albumen." The albuminuria existed before death, and may have existed even before admission to hospital. If we grant, as the author asks, that the kidneys became acutely diseased during the progress of the rheumatism, the state of these organs is, alone, sufficient to explain the peculiar head trouble observed in these four cases; that is to say, the stupor, delirium, and coma were simply uræmic and no more. I question whether the large hard kidney could be produced in the short time of fourteen days in case I; or the large white kidney in sixteen days in case II. Dr. Da Costa thinks the impaired action of the kidneys is proof of the rheumatism attacking them.

"This case" (case I) "is a striking illustration of the occurence of cerebral symptoms after apparent convalescence. But it is chiefly valuable because it proves the lesion of the kidneys that may happen in acute rheumatism, and that did happen after the case had been actually sometime under observation, for the first records show that the urine was healthy. Moreover it shows in what manner exposure" (the patient had freely bathed) "may act, and explains the occurence of suddenly developed stupor passing into coma; and I suspect that nearly all the so-called apoplectic instances of cerebral rheumatism which have been placed on record, are of a nature which this case brings out, as indeed it suggests the explanation of many of the nervous phenomena of the malady."

"Here is another instance" (case II) "in which there was stupor as well as delirium and in which the examination, after death, showed the kidneys diseased, but not the brain." * * * "These cases" (cases I and II) "show, then, a remarkable coincidence in some of the symytoms, and tell us in what direction we are to look in instances of stupor, or stupor and delirium arising in the course of cerebral rheumatism, before we seek for the explanation elsewhere."

Prof. Da Costa tells us he saw cerebral rheumatism arise under very opposite plans of treatment, and even where no treatment is adopted. He scouts the idea of any form of treatment or any drug being the *cause;* yet, from his words, I infer that a strong suspicion lurked in his mind that certain drugs could play the part of *causation*, for he says: "I notice it in several of my cases which were treated with bromides; I see it coming on with and coming on without treatment, and the inference, therefore, is, that as it arises on such opposite therapeutical plans no drug is responsible; though of course it is evident that with certain symptoms arising some drugs had better be avoided or discontinued." No "opposite therapeutical plans" were tried in these cases, and no case was left without treatment. Indeed all received the same indentical treatment except case IX, who was a private patient under another practitioner's care.

He believes " the true pathology of the cerebral disorder is to be sought in the action of the rheumatic poison on the brain, whether it does so directly or indirectly, through the changed composition of the blood or by both concurring. To these elements is often added an altered condition of the finer vessels of the brain. In other words the rheumatic poison may fasten upon the lining coat of these as it does upon the endo-cardium, and, favored by the altered condition of the blood, lead to plugging, as was so distinctly found in Case IV, and as was beginning in Case III. Occasionally it may be an embolus washed from the heart into the cerebral vessels that occasions the circulatory disorder, but more usually it is a thrombus there formed; and if in any case there be not actual obstruction, there is still circulation of altered blood, which, moreover, is apt to be still more vitiated by the impaired action of the kidneys—itself another expression of the rheumatic poison seizing upon an internal organ. The common condition of the brain tissue in cerebral rheumatism is that of nutrition interfered with, and of anæmia; and where rheumatic endocarditis or pericarditis coexist, this may show itself all the more quickly."

"A peculiar modification of meningitis occasionally sets in during the progress of acute rheumatism. The invasion is sudden, is accompanied by great fear of impending death, is followed by delirium, and often ends fatally—sometimes very rapidly so.'" — Tanner, Pract. Med., Sixth Ed., Vol. 1, page 345.

M. Bouchert says: "The serious complication of acute articular rheumatism called cerebral rheumatism is only—as proved by pathological anatomy and by the opthalmoscoscope—a form of meningitis. Examination of the membranes of the brain reveals a considerable venous stasis with an opaline infiltration of pia mater, caused by numerous leucocytes * * * * Rheumatism of the brain is ushered in by delirium more or less violent, terminating by coma or by asphyxia, sometimes very rapid which may cause death in a few hours."

There is no cellular tissue in the brain upon which the rheumatic poison can "fasten." The membranes, particularly the dura mater, which anatomically most closely resembles the tissues usually attacked in acute articular rheumatism, are the structures, within the cranium, liable to be involved. Then we should have the *délire foudroyant* of Virchow—intense delirium, convulsions, followed by rapid coma and death. "Mild, good-natured delirium" with meningitis would be very pleasant indeed. That cerebral rheumatism is ever due to embolism of the cerebral vessels is extremely doubtful. Emboli washed into the cerebral vessels in size or number to give rise to symptoms cause, usually, sudden convulsions, loss of consciousness, etc. Such is what is sometimes seen after ligature of the common carotid artery. Such, too, are the symptoms observed by me in cases of undoubted embolism of the cerebral vessels. The convulsions, etc., are due to the sudden deprivation of blood to a considerable part of the brain—to temporary anæmia of that part with consequent loss of function. Why should embolism during the course of acute articular rheumatism give rise to different results?

To suppose the "lining coat" of the cerebral vessels may be attacked, as Dr. DaCosta asserts, like the endocardium, while the endothelium of the vessels of the rest of the body escapes, lacks proof. There is no difference between the endothelium of the cerebral vessels and that of the general system. How then can he account for the exemption in the latter vessels. Besides, there is no anatomical indentity in the endothelium and in the endocardium. Can the poison attack the brain tissue proper, and not give evidence, post-mortem, of such implication? We have altered blood in all cases of rheumatism, yet how seldom do we see cerebral trouble.

In *diagnosis*, Dr. DaCosta considers it very important to distinguish the exact form of cerebral disturbance present. Some of those developed suddenly and formerly called apoplectic, he attributes to uræmia, while others are due to emboli washed from the heart into the cerebral vessels. Where hysterical delirium is marked, he thinks the condition is "plugging of the arteries of the brain from rheumatism affecting them, and not from heart disease." Where the delirium is fitful with a typhoid state, he thinks it is cerebral rheumatism with "anæmia of the brain." "Sometimes the symptoms are so truly typhoid that we ask ourselves whether such cases ought to be classed with cerebral rheumatism; would it not be better to regard them as 'typhoid rheumatism,' and to view the cerebral symptoms in the same light as we do those of the low fevers?" "In looking further at the diagnosis of cerebral rheumatism, we must not forget that the cardiac lesions may produce head symptoms."

The prognosis is very grave.

*Treatment.*—"Where we can, the treatment for the rheumatic symptoms must be continued, and in a form that shall not be depressant; hence I do not think the bromides ought, as a rule, be employed, which I did for a time, until I understood the meaning of the cerebral symptoms better." I might ask why the bromides were employed at all? Has bromide of ammonium —the salt employed by Dr. Da Costa—any curative value in acute rheumatism? Has any writer recommended its use in rheumatism? Not that I am aware. Nor do I think it possesses any curative power in such disease. It is depressant; it was not discontinued at all, but persistently employed to the last, even with Case XII, in which that drug was given until the patient appeared dead. It was then set aside and stimulants used liberally and boldly until the patient rallied and finally recovered under stimulants. Of stimulants, Dr. Da Costa thinks highly, and regrets not having used them more freely.

The treatment pursued in these eleven cases, when contrasted with the usual methods or with cases where no treatment is adopted, does not commend itself to others for imitation. In *twelve cases* "allowed to pursue their course under favorable hygienic circumstances, uninfluenced by therapeutic interference, the mean duration of the disease was twenty-six days, the minimum being twelve, and the maximum fifty-six days," says Dr. Austin Flint. In Dr. Da Costa's *eleven cases* (Case IX is omitted as being chronic) the minimum is sixteen days, the maximum sixty-five, with an average of forty-one days. This is far in excess of Dr. Flint's cases without treatment.

Closely examining these twelve cases of "cerebral rheumatism," the first was an instance, clearly, of Bright's disease in which hebetude, stupor, suppression of urine and death followed the usual course. The cause of death, as well as the peculiar "head symptoms" noted during the last illness, was explained by the discovery, post mortem, of the large, hard kidney. The brain was not examined. It is difficult to understand why the case is classed as one of cerebral rheumatism, when the pathological condition characteristic of Bright's disease was proved. Were not the delirium, stupor, and coma due to the state of the kidneys? And is it not just possible that the organic lesion of the kidneys was the cause of the rheumatism? This case, then, must be excluded.

In Case II there was albuminuria, granular and oily casts, and diminution of urine. Post mortem, the lungs were congested (hypostatic?); the kidneys large, pale, and in a state of fatty degeneration. This case, too, must be excluded, for it is another instance in which the peculiar cerebral condition can be attributed, and justly so, to the state of the blood owing to the kidney disease, as the autopsy so clearly proved. I think Prof. Da Costa goes out of his way to explain the "head symptoms" in these cases, on the hypothesis of cerebral rheumatism, when the explanation is given by the cases themselves in the organic disease of the kidneys. How does Dr. Da Costa diagnosticate these as instances of cerebral rheumatism? How does he differentiate between delirium, stupor, and coma occurring in the course of Bright's disease, and cerebral rheumatism, when his only data for calling these cerebral rheumatism were the stupor, delirium, and coma? Pneumonitis would appear to have complicated Case V. In Case VI also there was albuminuria on admission, with the pale, puffy face so indicative of Bright's disease. The kidneys were not examined microscopically. In Case VIII the urine was not tested during life; some drawn from the bladder after death was highly albuminous. Of course it was so before death. I think these two cases were likewise instances of organic lesion of the kidneys, and the delirium, stupor and coma were due to the state of these organs—to uræmia. In Case IX, Dr. Da Costa himself believes embolism of some cerebral vessels was the particular form of intra-cranial lesion present. There is insanity in the family; the patient, a lady subjected to severe trials, became insane, in which condition Dr. Da Costa saw her in consultation. Surely this case is not entitled to be called cerebral rheumatism. Case XI manifested "no cerebral symptoms" whatever. I cannot understand why the case is given in this paper at all.

Excluding, then, Cases I, II, VI, VIII, IX and XI as not being "cerebral rheumatism" at all, we have six cases which closely resemble each other. All were instances of acute, articular rheumatism receiving the same treatment. Taking the entire eleven cases treated in hospital, eight died and three recovered. Of the eight who died the brain was not examined in two; the brain alone was examined in two; no autopsy was permitted in one; and a general post mortem examination was made in three. Dr. DaCosta himself admits the autopsies were not as carefully made as they should be. Deduction from partial examinations cannot, obviously, be reliable nor valuable. As far as they went they revealed no organic disease of the brain.

The eleven patients were treated with bromide of ammonium, a neutral salt, and given in doses of twenty grains every two, three or four hours. It is quite remarkable that, though all the patients were several days—a few even weeks—sick before coming into hospital, yet nevertheless, neither delirium, stupor nor coma appeared before admission. These symptoms invariably appeared after the use of the bromide, in from four days the shortest, in a feeble-minded female, to twenty-four days the longest period after its exhibition had been commenced. This I consider a very important fact and the key to the whole trouble. It is also remarkable that in the cases in which the use of the bromide was persevered with, all died; that when it was discontinued at a comparatively early stage of the delirium, etc., as in Cases V, XI and XII, and acetate of potass., although only a remote, alkaliac remedy, substituted, the urine was soon found alkaline and maintained so by the use of the same drug, all three cases recovered. Stimulants, no doubt, contributed their share towards recovery.

Out of the eleven cases no less than six manifested some pulmonary trouble or complication. In Case II the lungs were congested; in Case III there was dullness on percussion, feeble respiratory murmer and cough; in Case IV there was cough; in Case V rapid breathing, oppression in chest, "fine râles and faint friction at lower part of right lung; both lungs were congested." In Case VII there were sonorous râles in posterior thorax and hypostatic congestion found post mortem. Case XII had dullness and harsh breathing posteriorly.

Wakefulness, delirium, stupor, pulmonary complications, such as hurried respiration and congestion of the lungs and paralysis of the sphincters shown by involuntary micturition and defecation in bed, are prominent features of Dr. DaCosta's cases. When the nutrition of the brain is lowered, as occurs in anæmia of that organ, it is hardly necessary to say the mental, motor and sensory functions are all more or less interfered with or perverted; that the peculiar delirium, coma, etc., observed by Prof. DaCosta are just that kind which capillary anæmia of the brain produces. He himself had a suspicion that the delirium might be caused, in the manner stated, by the bromide of ammonium, for he says: "I notice it in several of my cases which were treated with bromides;" and thinks the bromide of ammonium is one of the drugs which "had better be avoided or discontinued" since it is a decided depressant. But what caused the anæmia of the brain? Was it the bromide of ammonium? I think so.

Garrod, Meuriot, Amory, and especially Brown-Sequard have concluded from their experiments, "that bromides contract all the blood-vessels, producing anæmia of the brain and spinal cord and so diminishing the excitability of these organs." Experiments show that the bromides are vaso-motor stimulants, especially to the capillaries, and that the contraction of these vessels is due to the stimulation of the vaso-motor system. Bromides affect the reflex function of the cord only. They depress the sensory nerves, hence tickling, pinching and pricking of the soles of the feet give no evidence of sensation. They exercise a depressing influence upon the motor ganglia of the heart, lessen the frequency and force of the contractions of the heart and finally arrest it in diastole. "Diminished sensibility, followed by complete anæsthesia of the soft palate, uvula and upper part of the pharynx is the first symptom that the patient is getting under the influence of the drug," says Bazaire. Bromides induce general complete paralysis; they render patients low-spirited. Dr. Clark and others consider the well-known hypnotic effect of the bromides is due to the anæmia of the brain induced by these salts. Too much anæmia, Dr. Clark tells us, after experiments on himself, induces wakefulness, while a less degree produces sleep. It is well known that bromide of potassium produces an acneform rash, eczema, and spots resembling erythema nodosum.

To my mind Prof. DaCosta's cases show very clearly the physiological and the toxicological effects of the bromide of ammonium. The restlessness, sleeplessness, palsies, delirium, stupor and coma, except where due to organic disease of the kidneys as already pointed out, were due, I think, to the persistent use, in full and frequent doses, of the bromide of ammonium. I cannot, therefore, agree with the distinguished professor that the cases published were "cerebral rheumatism" at all. Bromide of ammonium is, in my opinion, clearly contra-indicated in acute articular rheumatism, since it is a powerful depressant, and possesses neither curative nor palliative value in that disease.

We may thus sum up the action cf the bromides. They act as local sedatives, diminishing and, in large doses, arresting muscular action and irritability. By direct contact they lower and paralyze nervous excitability in general, but more especially that of the glosso-pharyngeal nerves. They weaken and finally arrest the respiratory movements. They lower the action of the heart in a remarkable manner; cause diminution of the caliber of the capillary vessels, and, through the capillaries, act on all organs and tissues supplied by these vessels. They are eliminated through the saliva and urine and also through the skin in lesser quantity, giving rise to an erythematous eruption. In fine, their chief action being that of a vaso-motor stimulant, and such action being produced by direct contact, their effects will be chiefly manifested in those organs with which their contact will be most prolonged, that is, those through which they are eliminated. Hence their special action on the throat and on the urino-genital organs.

DR. M. O. JONES, for the last four years a resident practitioner in this city, has removed to his old home in Pittsburgh, Pa. During his residence here he won the confidence and esteem of all with whom he became acquainted.

